# A visualized model for identifying optimal candidates for aggressive locoregional surgical treatment in patients with bone metastases from breast cancer

**DOI:** 10.3389/fendo.2023.1266679

**Published:** 2023-10-05

**Authors:** Yuexin Tong, Shaoqing Xu, Liming Jiang, Chengliang Zhao, Dongxu Zhao

**Affiliations:** ^1^ Department of Orthopedics, The China-Japan Union Hospital of Jilin University, Changchun, Jilin, China; ^2^ Department of Orthopedics, Affiliated Hospital of Qingdao University, Qingdao, Shandong, China

**Keywords:** breast cancer, bone metastasis, primary tumor resection, nomogram, prognosis

## Abstract

**Background:**

The impact of surgical resection of primary (PTR) on the survival of breast cancer (BC) patients with bone metastasis (BM) has been preliminarily investigated, but it remains unclear which patients are suitable for this procedure. Finally, this study aims to develop a predictive model to screen BC patients with BM who would benefit from local surgery.

**Methods:**

BC patients with BM were identified using the Surveillance, Epidemiology, and End Results (SEER) database (2010 and 2015), and 39 patients were obtained for external validation from an Asian medical center. According to the status of local surgery, patients were divided into Surgery and Non-surgery groups. Propensity score matching (PSM) analysis was performed to reduce selection bias. Kaplan-Meier (K-M) survival and Cox regression analyses were conducted before and after PSM to study the survival difference between the two groups. The survival outcome and treatment modality were also investigated in patients with different metastatic patterns. The logistic regression analyses were utilized to determine significant surgery-benefit-related predictors, develop a screening nomogram and its online version, and quantify the beneficial probability of local surgery for BC patients with BM. Receiver operating characteristic (ROC) curves, the area under the curves (AUC), and calibration curves were plotted to evaluate the predictive performance and calibration of this model, whereas decision curve analysis (DCA) was used to assess its clinical usefulness.

**Results:**

This study included 5,625 eligible patients, of whom 2,133 (37.92%) received surgical resection of primary lesions. K-M survival analysis and Cox regression analysis demonstrated that local surgery was independently associated with better survival. Surgery provided significant survival benefits in most subgroups and metastatic patterns. After PSM, patients who received surgery had a longer survival time (OS: 46 months *vs*. 32 months, p < 0.001; CSS: 50 months *vs*. 34 months, p < 0.001). Logistic regression analysis determined six significant surgery-benefit-related variables: T stage, radiotherapy, race, liver metastasis, brain metastasis, and breast subtype. These factors were combined to establish the nomogram and a web probability calculator (https://sunshine1.shinyapps.io/DynNomapp/), with an AUC of 0.673 in the training cohort and an AUC of 0.640 in the validation cohort. The calibration curves exhibited excellent agreement. DCA indicated that the nomogram was clinically useful. Based on this model, surgery patients were assigned into two subsets: estimated sur-non-benefit and estimated sur-benefit. Patients in the estimated sur-benefit subset were associated with longer survival (median OS: 64 months *vs*. 33 months, P < 0.001). Besides, there was no difference in survival between the estimated sur-non-benefit subset and the non-surgery group.

**Conclusion:**

Our study further confirmed the significance of local surgery in BC patients with BM and proposed a novel tool to identify optimal surgical candidates.

## Introduction

Breast cancer (BC) is the leading cause of female mortality worldwide. Approximately 290560 new cases were diagnosed, and 43780 deaths in the United States alone in 2022 (1). Despite great advances in the systemic treatment of BC over recent years, largely attributable to the rise of endocrine therapy, anti-HER2 therapy, CDK4/6 inhibitor, and mTOR inhibitor (2), tumor progression and distant metastasis (DM) remain the major obstacles for the long-term survival of BC patients (3). Approximately 3.5–6% of newly diagnosed BC cases have synchronous metastasis, with bone being the most common site (4, 5). Approximately three-quarters of stage IV BC patients have bone metastases (BM). BC patients with BM usually experience a poor prognosis, with a three-year survival of 25% and a five-year survival rate of 13% (6).

Generally, BC presented with BM is considered a virtually incurable disease, with therapeutic goals primarily focusing on symptom relief and quality of life (7). Whether these patients should undergo surgical intervention for primary lesions remains controversial. The conventional view believed that locoregional surgery for BC patients with metastatic disease reduced only the local tumor burden without preventing the disease’s progression. It would also expose the patients to surgical risks and post-operative complications (8–10). Recent reports suggest that the surgical resection of primary tumors improves survival in BC patients with BM. A large cohort retrospective study designed by Huang et al. revealed that BC patients with BM who underwent local surgery had better survival outcomes than those who did not (median survival: 50 months versus 31 months, p < 0.001) (11). Another study demonstrated that local surgery positively impacted survival in BC patients with BM (12). However, this work was limited to investigating the significance of surgery for BC patients with only BM. The disparity of surgical benefits among different patterns of synchronous extraskeletal metastases, such as the lung, brain, and liver, in BC patients with BM at initial diagnosis has not been thoroughly studied. Individual patient characteristics and differences in disease progression make it unclear whether primary tumor resection should be recommended for all BC patients with synchronous BM.

Surgeons are frequently challenged in their daily practice to make reasonable judgments about the suitability of BC patients with synchronous BM for locoregional surgery based on the understanding of the vast amount of high-dimensional and heterogeneous data combined with their knowledge of clinical experience to inform their clinical decisions. Poor medical decisions may not provide the best treatment options, affecting patient safety and increasing healthcare costs (13). A nomogram, which has been developed for various malignancies, can provide visualized probability estimates tailored to each individual as an easy-to-use and integrated prediction model (14, 15). To date, no such model exists to estimate the probability of local surgical benefit in BC patients with BM and thus to guide rational medical decisions for surgical treatment in this population. Hence, we used a population-based database to address the clinical needs and thoroughly explore the role of local surgery in BC patients with BM. We propose a novel prediction model that accurately identifies patients who could benefit from surgical resection of primary lesions.

## Methods

### Study population

Research data was extracted from the Surveillance, Epidemiology, and End Results (SEER) database using the Client-Server Mode of SEER*Stat 8.1.4 software. A signed SEER research data agreement was submitted to the SEER program for approval to access the database. This study was conducted per the Declaration of Helsinki (revised in 2013). SEER data can be used publicly for cancer-based epidemiological research because they do not contain personal identification information. Thus, our study was exempt from ethical review and informed consent. Since the definite metastasis site was only recorded from 2010 onward, patients with histologically confirmed BC (Primary Site-labeled: C50.0-C50.9) with bone metastases from 2010 to 2015 were retrospectively reviewed in our study. Subsequently, cases were excluded if they met the following criteria: (a) demographic, clinicopathological, and treatment variables selected for our analysis were unknown; (b) BC was not the first primary malignant tumor; (c) survival month less than one month; and (d) patients with a diagnosis according to clinical or imaging findings or autopsy. Ultimately, 5,625 eligible patients were identified from 13,048 BC patients with BM to constitute the initial analysis cohort. Furthermore, the external validation set data, including 39 patients, was obtained from the Affiliated Hospital of Qingdao University. Two orthopedic surgeons were assigned to record clinical, pathological, and therapeutic information on the patient using a blinded method.

**Figure 1:**
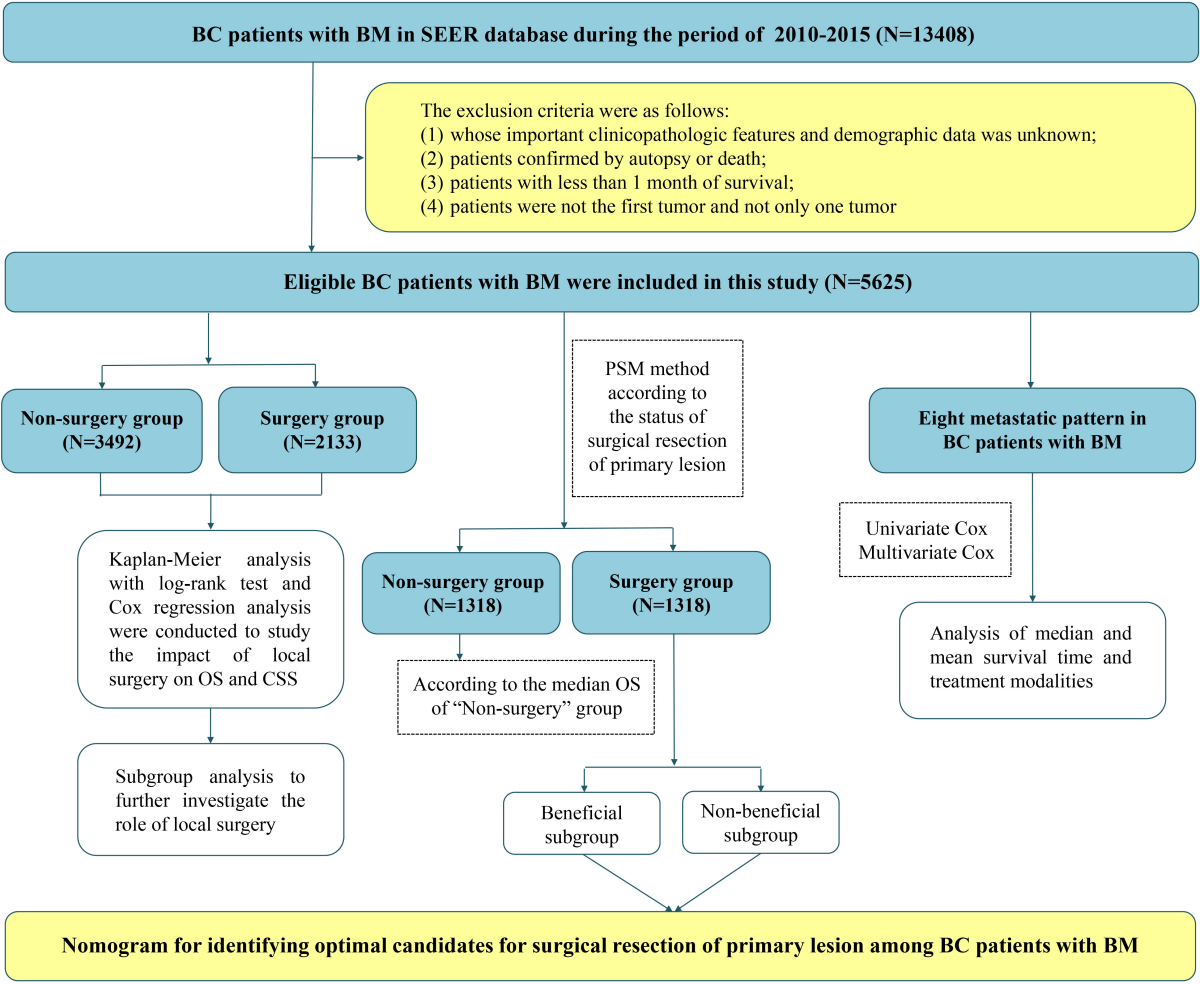
The patient selection and workflow of this study.

Besides, demographic information (age, gender, and race), tumor characteristics (histology, primary site, laterality, breast subtype, grade, T stage, N stage, tumor size, liver metastasis, lung metastasis, and brain metastasis), treatment modalities (radiotherapy, chemotherapy, surgery to DM, and surgery), survival time, and vital status variables in BC patients with BM were collected in this study. The primary site was defined according to the International Classification of Diseases for Oncology (ICD-O) codes: central portion (C50.1), upper-inner (C50.2), lower-inner (C50.3), upper-outer (C50.4), lower-outer (C50.5), and others (C50.0, C50.6, C50.8, and C50.9). The histology was classified as invasive ductal carcinoma (IDC), invasive lobular carcinoma (ILC), and others. Continuous variables, age at diagnosis and tumor size, were transformed into categorical variables (age: < 60 years and ≥ 60 years; tumor size: < 5 cm, 5–10 cm, and >10 cm). Surgery was defined as direct cancer surgery on the primary tumor, including partial breast-conserving surgery, radical mastectomy, modified radical mastectomy, and local tumor resection. Months of survival, vital status records, and cause-specific death classification were employed to calculate overall survival (OS) and cancer-specific survival (CSS). The patient selection and workflow of this study is illustrated in **Figure 1**.

### Statistical analysis

BC Patients with BM were divided into surgery and Non-surgical groups according to whether they underwent surgery at the primary tumor site. The propensity score matching (PSM) method was performed to minimize selection bias and the influence of potential confounding factors. Patients in two groups (Surgery and Non-surgery) were matched at a ratio of 1:1 using the closest propensity score on the logit scale with a caliper value of 0.001. The chi-square test or Fisher’s exact test was utilized to compare the differences in selected variables between two groups before and after PSM analysis. Subsequently, differences in OS and CSS were explored between the two patient groups using Kaplan-Meier (K-M) analysis with a log-rank test in the initial and matched cohorts. Additionally, the predictive value of locoregional surgery was evaluated using univariate and multivariate analyses based on Cox proportional hazard models. A two-sided P-value of < 0.05 was considered statistically significant. All data were analyzed using SPSS 26.0 (IBM Corporation, Armonk, NY, USA) software and R version 4.1.1 (http://www.r-project.org/).

### Establishment and validation of a web-based nomogram to estimate the probability of benefit from local surgery in BC patients with BM

This study assumed that patients who received local surgery and had longer survival than patients who did not could benefit from surgical intervention. Based on this hypothesis, in the matched cohort, Surgery group patients were classified as benefiting from surgery and not benefiting from surgery based on the median OS of patients in the Non-surgery group. Initially, patients who received surgery were randomly divided into a training and a validation cohorts in a 7:3 ratio. Subsequently, the univariate and multivariate logistics regression analyses were conducted to determine the independent surgery-benefit-related variables among BC patients with BM. Simultaneously, a nomogram incorporating significant predictors was constructed using R software with the “rms” package to estimate the beneficial probability of locoregional surgery and select optimal candidates. Furthermore, a web-based nomogram was constructed using the “DynNom” package and the resulting survival probability formula to accurately calculate the beneficial probability of surgery in BS patients with BM.

The total point for each participant who underwent local surgery was calculated to verify the predictive accuracy of this newly developed model. The predicted beneficial probabilities of benefit were obtained from surgical resection of the primary lesion based on the nomogram. Afterward, patients with a predicted probability of benefit greater than 50% were assigned to the estimated sur-benefit subset, while those with less than 50% were assigned to the estimated sur-non-benefit subset. The difference in OS status between these patients and those who did not undergo surgery was investigated using K-M survival analysis using a log-rank test to verify whether the novel nomogram could appropriately screen the most suitable BC patients with BM for locoregional surgery. Additionally, receiver operating curves (ROC) and the area under the curve (AUC) were performed to assess the discriminative ability and calibration of the nomogram. The calibration curves plotted from 1,000 times bootstrap resampling using the “calibrate” function in the “rms” package were conducted to verify the agreement between predicted values and actual observations. The decision curve analysis (DCA) was used to evaluate the clinical usefulness of the novel model by quantifying the net benefits at different threshold probabilities.

## Result

### Patient baseline characteristics before and after PSM

This study included eligible 5,625 BC patients with bone metastases. Among these, 2,133 (37.92%) patients received surgical resection of primary lesion referred to as the Surgery group, whereas the remaining 3,492 (60.08%) patients were referred to the Non-surgery group. **Table 1** summarizes the patient’s demographic information, tumor characteristics, and treatment options. Race, grade, T stage, N stage, lung metastasis, liver metastasis, brain metastasis, radiotherapy, and chemotherapy varied significantly between the two groups. After a 1:1 PSM analysis, the matched cohort consisted of 2,636 BC patients with BM, including 1,318 patients in the Surgery group and 1,318 patients in the Non-surgery group. Chi-square test results revealed that baseline data of patients were balanced in the two groups (all P > 0.05, **Table 1**).

**Table 1:** Clinicopathological characteristics for BC patients with BM between 2010 and 2015 from the SEER database.

Variables	Before PSM				After PSM			
	Overall (n=5625, %)	Non-surgery (n=3492, %)	Surgery (n=2133, %)	p-value	Overall (n=2636, %)	Non-surgery (n=1318, %)	Surgery (n=1318, %)	p-value
**Age**				0.327				0.5589
< 60 years	2867(50.97)	1762 (50.46)	1105 (51.80)		1344 (50.99)	680 (51.59)	664 (50.38)	
≥ 60 years	2758(49.03)	1730 (49.54)	1028 (48.20)		1292 (49.01)	638 (48.41)	654 (49.62)	
**Race**				0.580				0.1382
African American	905(16.09)	572 (16.38)	333 (15.61)		417 (15.82)	191 (14.49)	226 (17.15)	
Other	443(7.88)	281 (8.05)	162 (7.59)		186 (7.06)	90 (6.83)	96 (7.28)	
Caucasian populations	4277(76.04)	2639 (75.57)	1638 (76.79)		2033 (77.12)	1037 (78.68)	996 (75.57)	
**Sex**				0.028				1.000
Female	5540(98.49)	3449(98.77)	2091(98.03)		2607 (98.90)	1303 (98.86)	1304 (98.94)	
Male	85(1.51)	43(1.23)	42(1.97)		29 (1.10)	15 (1.14)	14 (1.06)	
**Laterality**				0.594				0.7851
Left	2899(51.5)	1790(51.26)	1109(51.99)		1334 (50.61)	663 (50.30)	671 (50.91)	
Right	2726(48.5)	1702(48.74)	1024(48.01)		1302 (49.39)	655 (49.70)	647 (49.09)	
**Grade**				<0.001				0.7137
I	501 (8.91)	338(9.68)	163(7.64)		226 (8.57)	108 (8.19)	118 (8.95)	
II	2650 (47.11)	1765(50.54)	885(41.49)		1198 (45.45)	613 (46.51)	585 (44.39)	
III	2454 (43.63)	1372(39.29)	1082(50.73)		1208 (45.83)	595 (45.14)	613 (46.51)	
IV	20 (0.36)	17(0.49)	3(0.14)		4 (0.15)	2 (0.15)	2 (0.15)	
**T stage**				0.0001				0.1984
T1-T2	2848 (50.63)	1697(48.60)	1151(56.97)		1356 (51.44)	661 (50.15)	695 (52.73)	
T3-T4	2777 (49.37)	1795(51.40)	982(46.03)		1280 (48.56)	657 (49.85)	623 (47.27)	
**N stage**				<0.001				0.4935
N0	1230 (21.87)	884(25.32)	346(16.22)		521 (19.76)	253 (19.20)	268 (20.33)	
N1-3	4395 (78.13)	2608 (74.68)	1787 (83.78)		2115 (80.24)	1065 (80.80)	1050 (79.67)	
**Primary site**				0.523				0.2589
Central portion of breast	430 (7.64)	253(7.25)	177(8.30)		198 (7.51)	85 (6.45)	113 (8.57)	
Lower-inner	231 (4.11)	147(4.21)	84(3.94)		100 (3.79)	52 (3.95)	48 (3.64)	
Lower-outer	323 (5.74)	200(5.73)	123(5.77)		139 (5.27)	71 (5.39)	68 (5.16)	
Upper-inner	420 (7.47)	250(7.16)	170(7.97)		185 (7.02)	89 (6.75)	96 (7.28)	
Upper-outer	1541 (27.40)	974(27.89)	567(26.58)		771 (29.25)	405 (30.73)	366 (27.77)	
Other	2680 (47.64)	1668(47.77)	1012(47.44)		1243 (47.15)	616 (46.74)	627 (47.57)	
**Histology**				0.270				0.2835
Ductal	4295 (76.36)	2691(77.06)	1604(75.20)		2019 (76.59)	1023 (77.62)	996 (75.57)	
Lobular	625 (11.11)	374(10.71)	251(11.77)		291 (11.04)	133 (10.09)	158 (11.99)	
Other	705 (12.53)	427(12.23)	278(13.03)		326 (12.37)	162 (12.29)	164 (12.44)	
**Breast subtype**				0.161				0.866
HR+/HER2-	3780 (67.20)	2364(67.70)	1416(66.39)		1812 (68.74)	898 (68.13)	914 (69.35)	
HR+/HER2+	969 (17.23)	609(17.44)	360(16.88)		410 (15.55)	211 (16.01)	199 (15.10)	
HR-/HER2+	348 (6.19)	215(6.16)	133(6.24)		162 (6.15)	84 (6.37)	78 (5.92)	
HR-/HER2-	528 (9.39)	304(8.71)	224(10.50)		252 (9.56)	125 (9.48)	127 (9.64)	
**Tumor size**				0.335				0.7707
< 5 cm	3308 (58.81)	2048(58.65)	1260(59.07)		1553 (58.92)	770 (58.42)	783 (59.41)	
5-10 cm	1972 (35.06)	1241(35.54)	731(34.27)		919 (34.86)	468 (35.51)	451 (34.22)	
> 10 cm	345 (6.13)	203(5.81)	142(6.66)		164 (6.22)	80 (6.07)	84 (6.37)	
**Lung metastasis**				<0.001				0.961
No	4280 (76.09)	2473(70.82)	1807(84.72)		2114 (80.20)	1056 (80.12)	1058 (80.27)	
Yes	1345 (23.91)	1019(29.18)	326(15.28)		522 (19.80)	262 (19.88)	260 (19.73)	
**Liver metastasis**				<0.001				0.3084
No	4413 (78.45)	2576(73.77)	1837(86.12)		2167 (82.21)	1073 (81.41)	1094 (83.00)	
Yes	1212 (21.55)	916(26.23)	296(13.88)		469 (17.79)	245 (18.59)	224 (17.00)	
**Brain metastasis**				<0.001				0.918
No	5298 (94.19)	3227(92.41)	2071(97.09)		2538 (96.28)	1270 (96.36)	1268 (96.21)	
Yes	327 (5.81)	265(7.59)	62(2.91)		98 (3.72)	48 (3.64)	50 (3.79)	
**Surgery to DM**				0.903				0.4796
No	5380 (95.64)	3339(95.62)	2041(95.69)		2534 (96.13)	1271 (96.43)	1263 (95.83)	
Yes	245 (4.36)	153(4.38)	92(4.31)		102 (3.87)	47 (3.57)	55 (4.17)	
**Radiotherapy**				<0.001				0.7205
No	3318 (58.99)	2261(64.75)	1057(49.55)		1580 (59.94)	785 (59.56)	795 (60.32)	
Yes	2307 (41.01)	1231(35.25)	1076(50.45)		1056 (40.06)	533 (40.44)	523 (39.68)	
**Chemotherapy**				<0.001				0.7532
No	2385 (42.40)	1634(46.79)	751(35.21)		1143 (43.36)	567 (43.02)	576 (43.70)	
Yes	3240 (57.60)	1858(53.21)	1382(64.79)		1493 (56.64)	751 (56.98)	742 (56.30)	

### The relationship between locoregional surgery and survival in BC patients with BM

**Figure 2:**
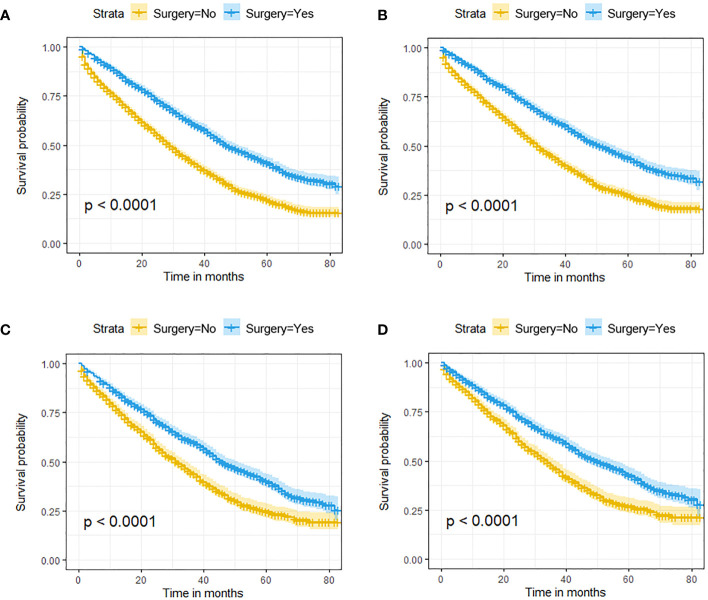
The effect of locoregional surgery on survival outcomes in BC patients with BM. Kaplan-Meier (K-M) survival curves for OS in the initial cohort **(A)** and the matched cohort **(B)** and for CSS in the initial cohort **(C)** and the matched cohort **(D)**.

**Figure 3:**
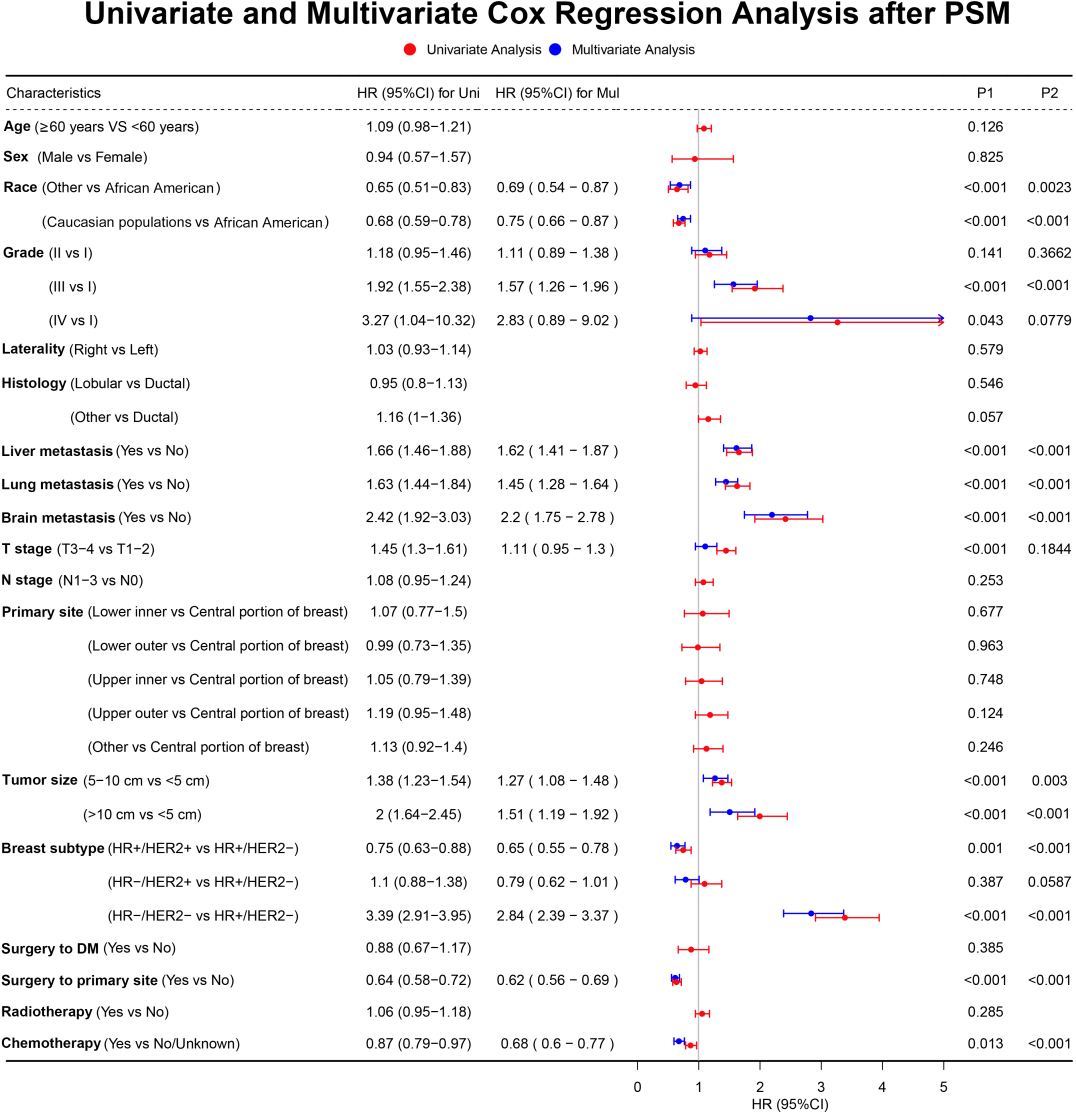
The forest plot for univariate and multivariate Cox regression analysis results.

**Figure 4:**
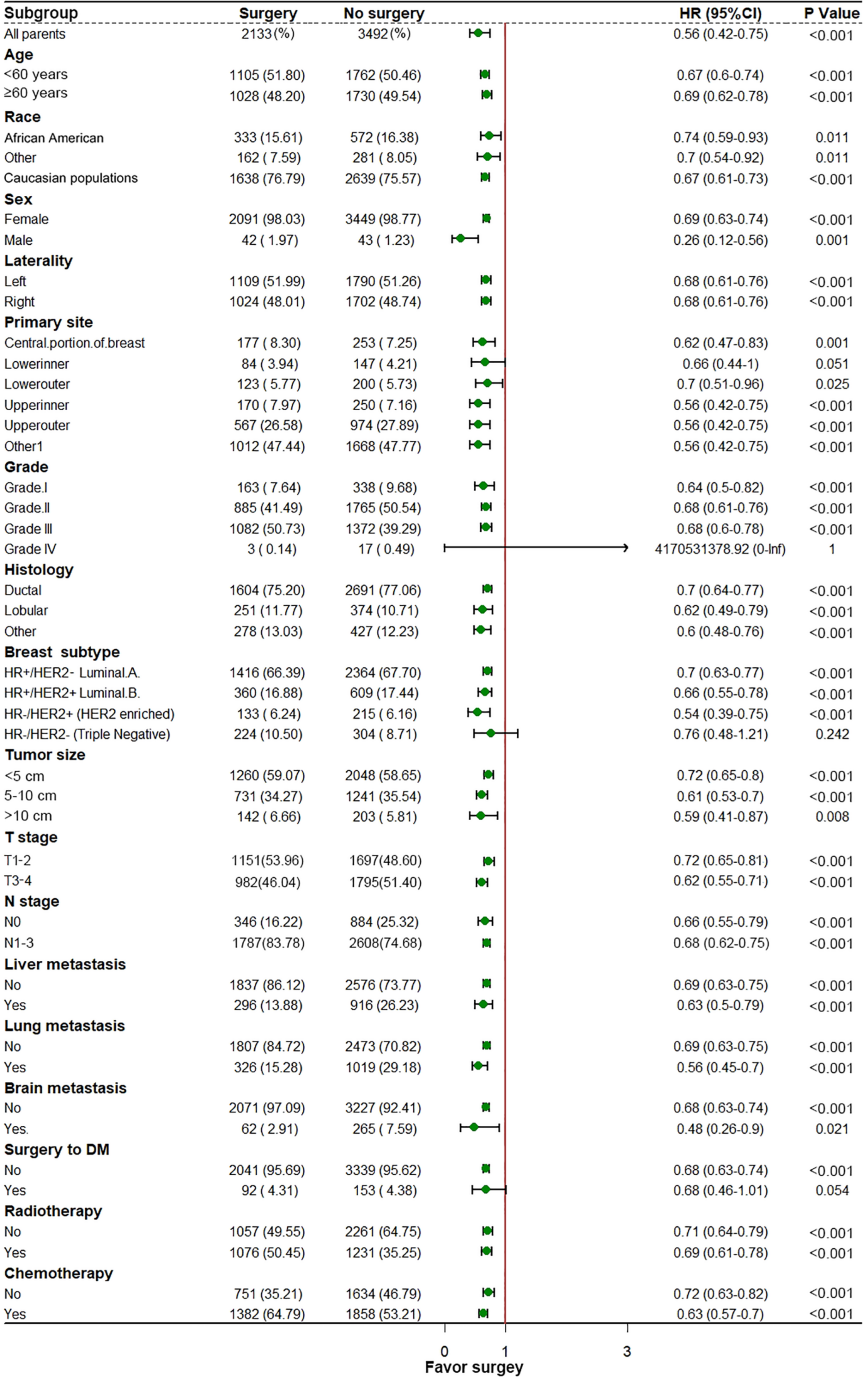
In the subgroup analysis for investigating the survival benefit of locoregional surgery in each specific patient’s group, the median dot represents the Hazard Ratio (HR), and the horizontal line represents a 95% confidence interval (95% CI).

In the initial cohort, BC patients with BM who received surgery had more satisfactory survival outcomes than those who did not. The K-M survival analysis indicated that patients in Surgery group had longer median OS and CSS (OS: 47 months [95% CI: 44.16–49.84]; CSS: 51 months [95% CI: 47.60–54.41]) than patients in Non-surgery group (OS: 29 months [95% CI: 27.65–30.35]; CSS: 31 months [95% CI: 29.41–32.59]). After the 1:1 PSM method, the significant survival benefit of locoregional surgery remained in the matched cohort. Specifically, the Surgery group had median OS and CSS of 46 months (95% CI: 42.44–49.56) and 50 months (95% CI: 45.61–54.39), whereas the Non-surgery group had 32 months (95% CI: 29.45–34.55) and 34 months (95% CI: 31.30–36.70) (**Figure 2**). Additionally, univariate and multivariate Cox regression analyses confirmed that surgery was an independent protective factor (HR: 0.57, 95% CI: 0.51–0.63, p < 0.001) for BC patients with BM (**Figure 3**). Besides, we performed subgroup analysis using the Cox hazard regression model to investigate further surgical resection’s impact on survival in specific subgroups. We discovered that locoregional surgery enabled improved OS for BC patients with BM in most subgroups (**Figure 4**).

### Survival and treatment modality for BC patients with BM at different metastatic patterns

**Figure 5:**
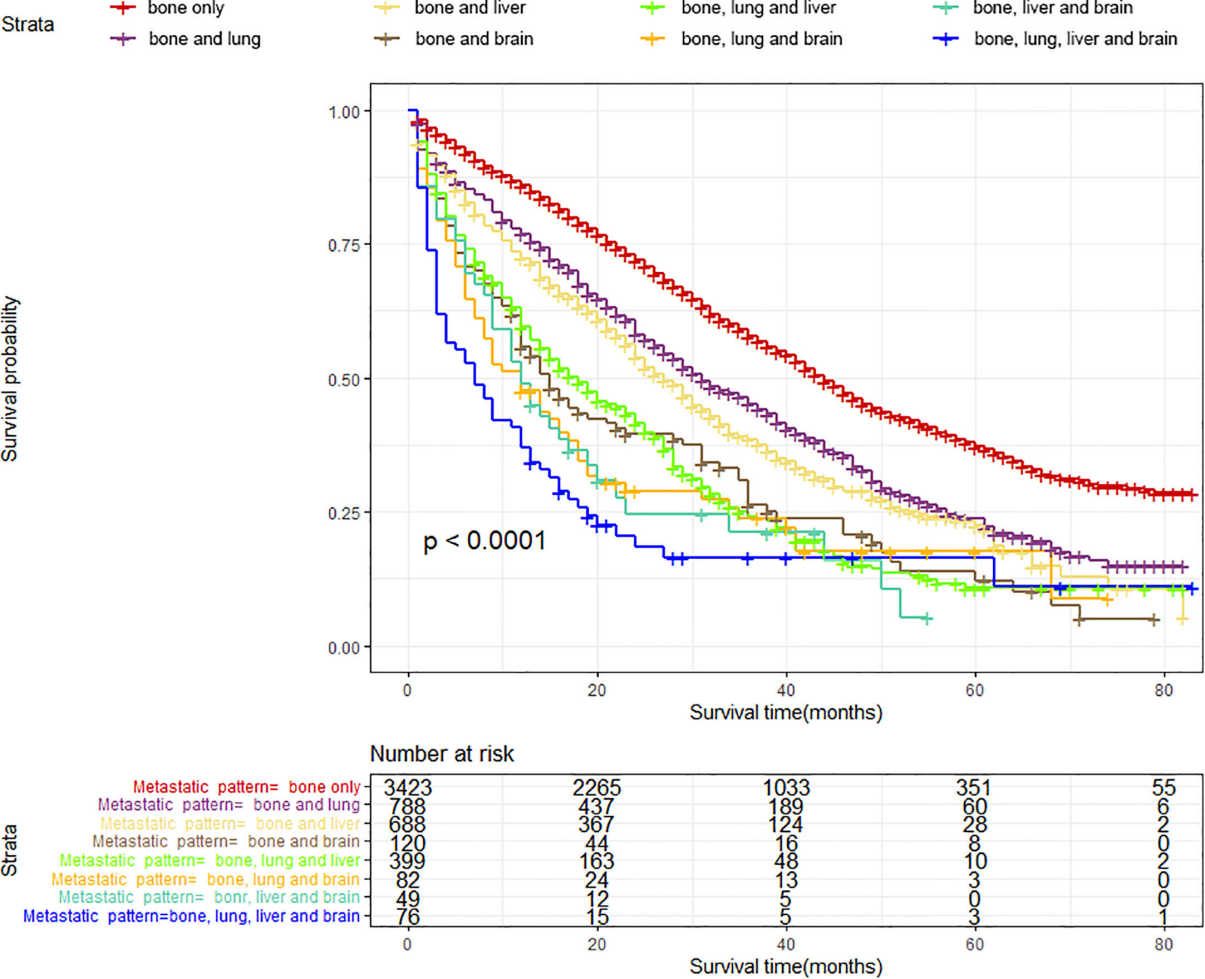
Kaplan–Meier survival curves with log-rank tests showed significant differences in OS among different metastatic patterns in BC patients with BM.

According to the site of synchronous extraskeletal metastasis, we classified the total cohort of BC patients with BM into eight metastatic patterns. The survival differences caused by different metastatic patterns in BC patients with BM were explored using the K-M survival analysis. **Table 2** and **Figure 5** reveal that patients with only BM have the most satisfactory median OS (44 months [95% CI: 42.08–45.92], while patients with bone, liver, lung, and brain metastases have the shortest median OS (7 months [95% CI: 3.58–10.42]). Patients with bone and brain metastases had the lowest survival rate among those with two metastatic sites. Among BC patients with BM accompanied by two synchronous extraskeletal metastases, the prognosis was dismal as long as the presentation of brain metastasis. Those patients with bone, lung, and brain metastases (Median OS: 12 months [95% CI: 7.27–16.73], Mean OS: 22.62 months [95% CI: 16.95–28.29]) had similar poor survival to those with bone, liver and brain metastases (Median OS: 12 months [95% CI: 8.08–15.92], Mean OS: 19.04 months [95% CI: 13.75–24.33]).

**Table 2:** The OS rates (median, mean) of BC patients with BM in different metastatic patterns.

Metastatic pattern	Median survival (months)	95%CI	Mean survival (months)	95%CI
Bone-only	44	42.08-45.92	46.97	45.83-48.12
Bone and lung	31	27.51-34.49	36.75	34.61-38.90
Bone and liver	27	24.58-29.42	33.45	31.09-35.80
Bone and brain	15	11.08-18.92	25.16	20.65-29.68
Bone, liver and lung	18	14.41-21.59	25.53	22.84-28.22
Bone, lung and brain	12	7.27-16.73	22.62	16.95-28.29
Bone, liver and brain	12	8.08-15.92	19.04	13.75-24.33
Bone, lung, liver and brain	7	3.58-10.42	18.83	12.43-25.43

**Table 3** summarizes the distribution of treatment modalities for BC patients with BM for each metastatic pattern. The multivariate Cox regression analysis results indicated that surgical resection of the primary lesion could provide survival benefit for BC patients with BM in more than half of metastatic patterns, including bone only (HR: 0.54, 95% CI: 0.49–0.61, p < 0.001), bone and lung (HR: 0.65, 95% CI: 0.53–0.80, p < 0.001), bone and liver (HR: 0.68, 95% CI: 0.55–0.84, p < 0.001), bone and brain (HR: 0.60, 95% CI: 0.37–0.98, p = 0.0405), as well as bone, liver, and lung (HR: 0.68, 95% CI: 0.50–0.92, p = 0.0125). Additionally, we observed that surgery, radiotherapy, and chemotherapy failed to significantly prolong the survival of patients in the metastatic pattern of bone, liver, and brain metastases, as well as bone, liver, lung, and brain metastases.

**Table 3:** Univariate and multivariate Cox regression analysis for OS based on treatment modalities and metastatic patterns in BC patients with BM.

Metastatic pattern(N)	Treatment	Status(N)	Univariate analysis HR (95% CI)	P value	Multivariate analysis HR (95% CI)	P value
	Surgery	No(1859)	0.57 (0.52-0.63)	<0.001	0.54 ( 0.49-0.61 )	<0.001
		Yes(1564)				
Bone-only(3423)	Radiotherapy	No(1958)	0.88 (0.8-0.97)	0.012	1.05 ( 0.95-1.16 )	0.3471
		Yes(1465)				
	Chemotherapy	No(1609)	0.68 (0.62-0.75)	<0.001	0.65 ( 0.58-0.73 )	<0.001
		Yes(1814)				
	Surgery	No(566)	0.69 (0.56-0.84)	<0.001	0.65 ( 0.53-0.8 )	<0.001
		Yes(222)				
Bone and lung(788)	Radiotherapy	No(524)	0.92 (0.76-1.11)	0.402		
		Yes(264)				
	Chemotherapy	No(371)	0.91 (0.76-1.09)	0.294		
		Yes(417)				
	Surgery	No(480)	0.73 (0.59-0.9)	0.003	0.68 ( 0.55-0.84 )	<0.001
		Yes(208)				
Bone and liver(688)	Radiotherapy	No(485)	1.02 (0.84-1.26)	0.815		
		Yes(203)				
	Chemotherapy	No(166)	0.58 (0.47-0.71)	<0.001	0.56 ( 0.44-0.71 )	<0.001
		Yes(522)				
	Surgery	No(87)	0.52 (0.32-0.84)	0.008	0.60 ( 0.37-0.98 )	0.0405
		Yes(33)				
Bone and brain(120)	Radiotherapy	No(26)	0.95 (0.57-1.58)	0.847	0.95 (0.57-1.58)	0.847
		Yes(94)				
	Chemotherapy	No(53)	0.72 (0.48-1.07)	0.106		
		Yes(67)				
	Surgery	No(322)	0.74 (0.56-0.99)	0.042	0.68 ( 0.5-0.92 )	0.0125
		Yes(77)				
Bone, liver and lung(399)	Radiotherapy	No(264)	1.05 (0.83-1.34)	0.658		
		Yes(135)				
	Chemotherapy	No(110)	0.60 (0.47-0.77)	<0.001	0.55 ( 0.42-0.72 )	<0.001
		Yes(289)				
	Surgery	No(64)	0.99 (0.55-1.77)	0.968		
		Yes(8)				
Bone, lung and brain(82)	Radiotherapy	No(24)	0.87 (0.51-1.49)	0.613		
		Yes(58)				
	Chemotherapy	No(34)	0.43 (0.26-0.71)	0.001	0.26 ( 0.15-0.47 )	<0.001
		Yes(48)				
	Surgery	No(47)	3.17 (0.74-13.53)	0.12		
		Yes(2)				
Bone, liver and brain(49)	Radiotherapy	No(18)	0.95 (0.49-1.84)	0.883		
		Yes(31)				
	Chemotherapy	No(14)	0.63 (0.32-1.23)	0.175		
		Yes(35)				
	Surgery	No(67)	1.03 (0.49-2.18)	0.931		
		Yes(9)				
Bone, lung, liver and brain(76)	Radiotherapy	No(19)	0.87 (0.48-1.56)	0.631		
		Yes(57)				
	Chemotherapy	No(28)	0.61(0.37-1.02)	0.057		
		Yes(48)				

### Construction and validation of nomogram to identify optimal surgical candidates among BC patients with BM

**Figure 6:**
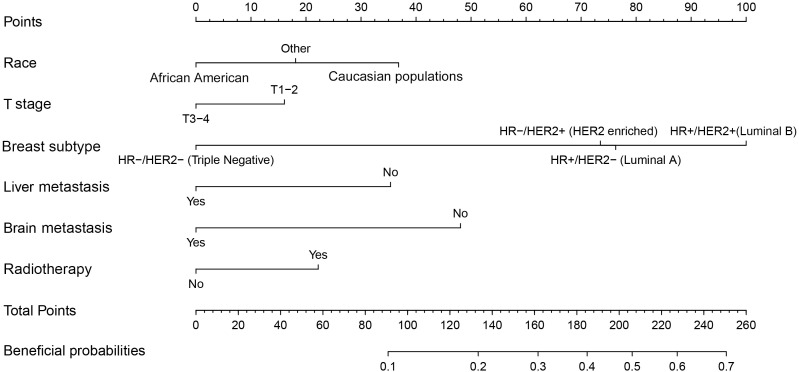
A screen nomogram to estimate the probability of surgical benefit and select optimal candidates for locoregional surgery in BC patients with BM.

**Figure 7:**
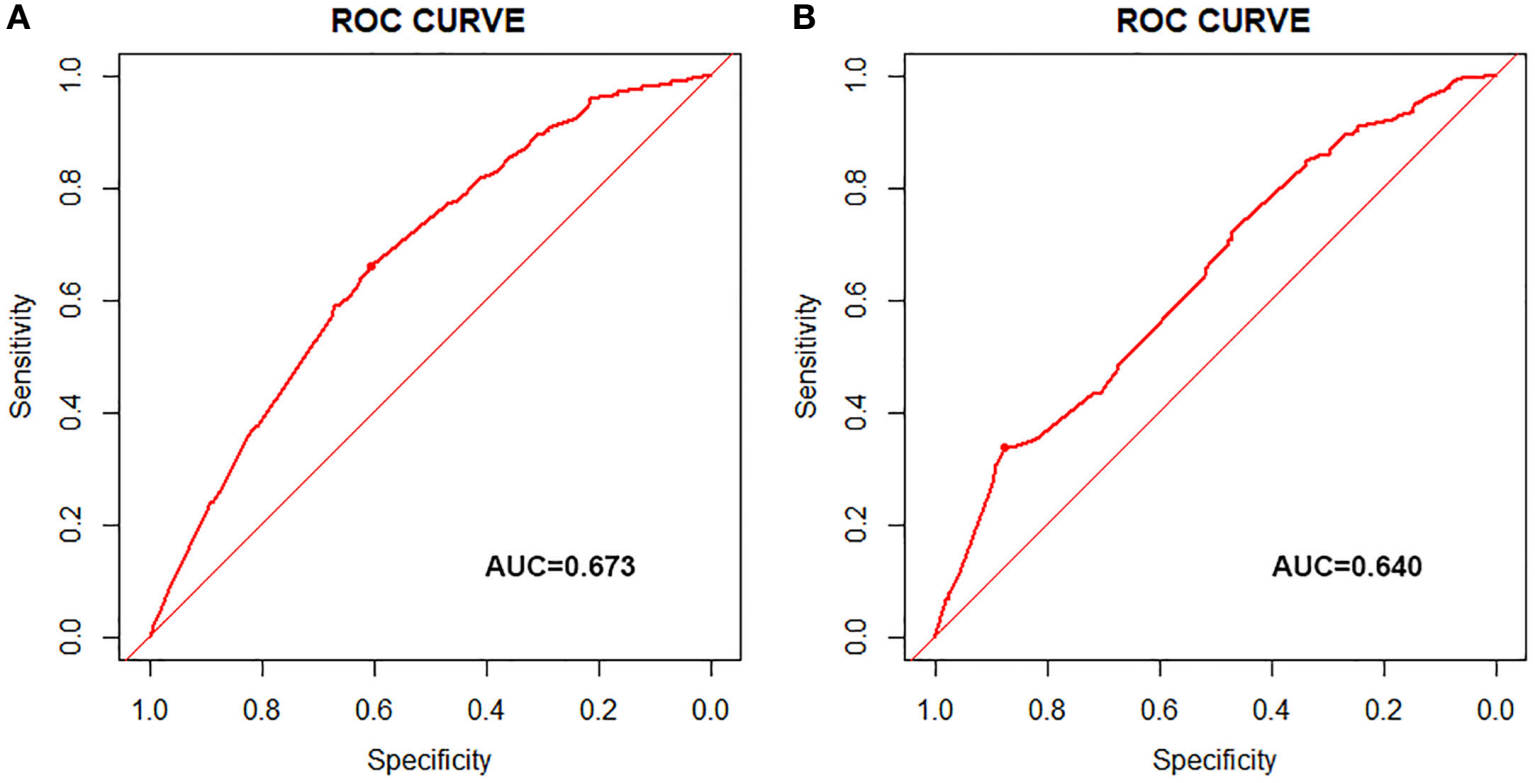
ROC curves in training cohort **(A)** and validation cohort **(B)**.

**Figure 8:**
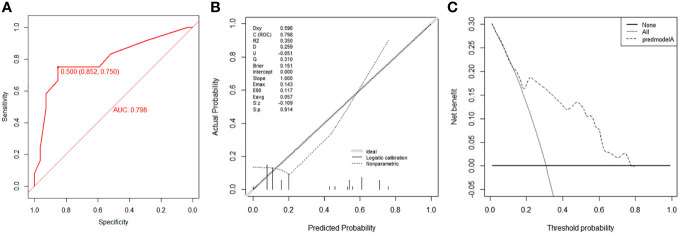
Validation of the nomogram using ROC curve **(A)**, calibration curve **(B)** and DCA **(C)** in the external validation cohort.

**Figure 9:**
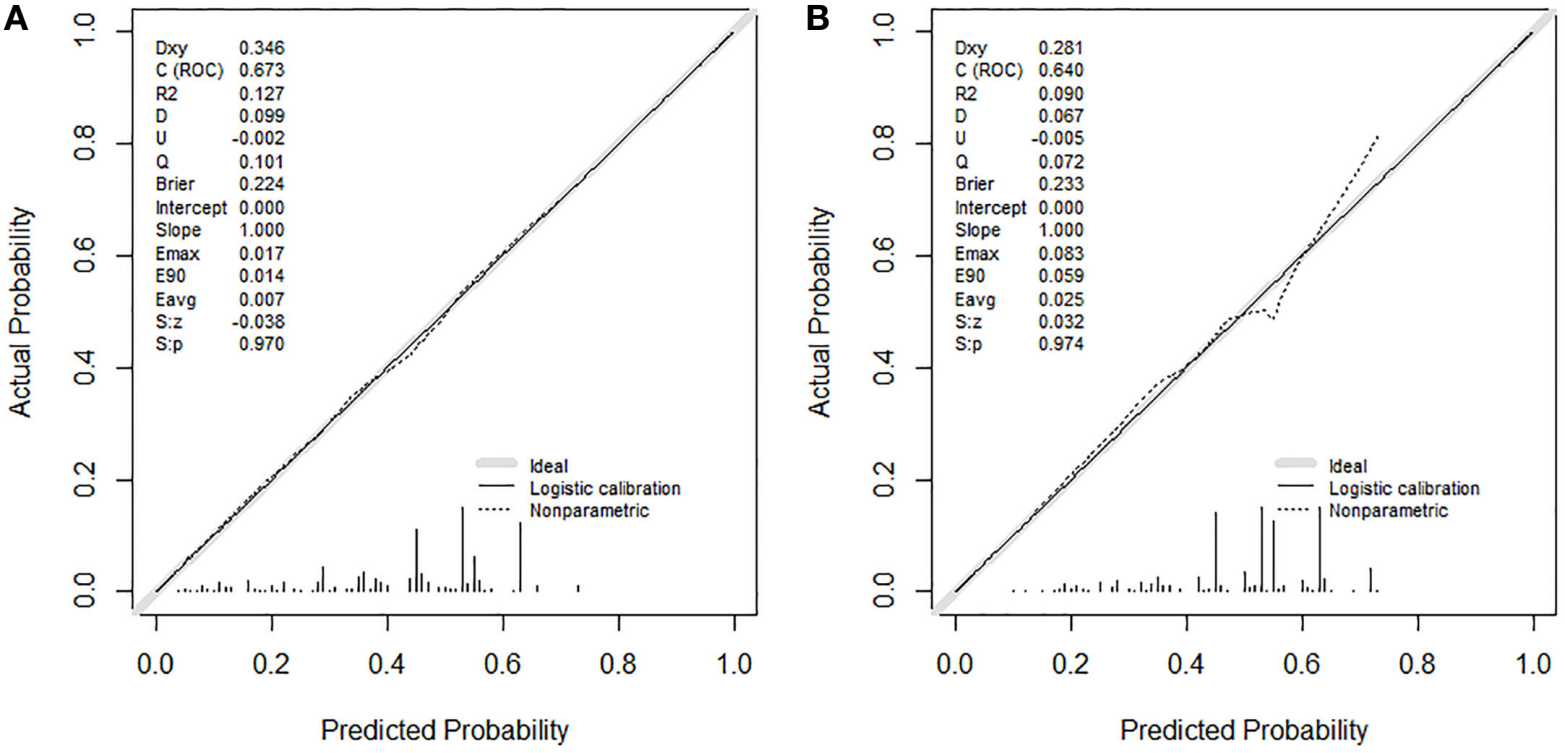
Calibration curves in training cohort **(A)** and validation cohort **(B)**.

**Figure 10:**
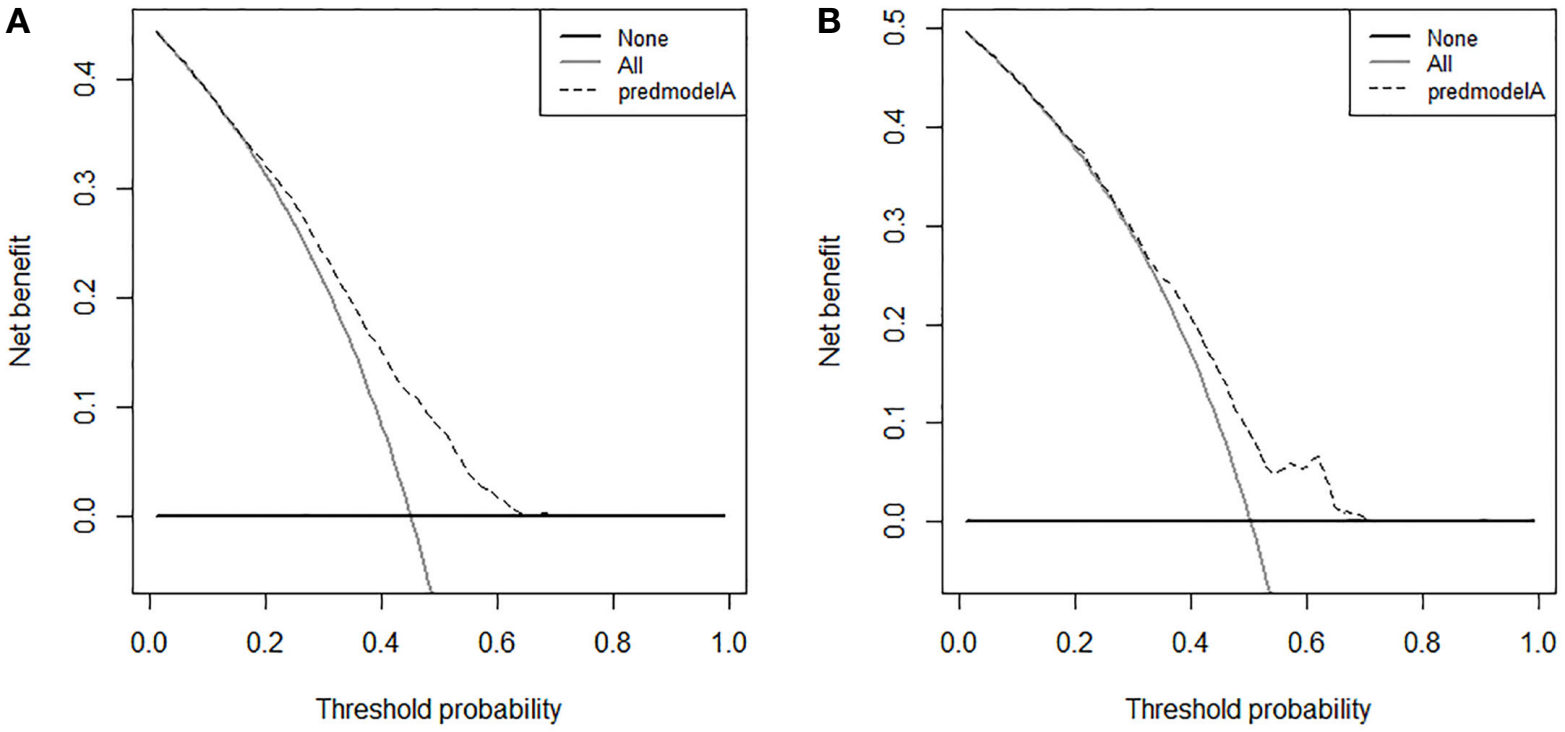
Decision curve analysis in training cohort **(A)** and validation cohort **(B)**.

According to the median OS (32 months) of patients in the Non-surgery group, patients who received locoregional surgery and survived for more than 32 months were defined as benefiting from surgery state (614, 46.59%), while those who survived for less than or equal to 32 months were defined as not benefiting from surgery state (704, 53.41%). All patient who received surgery (n=1318) were randomly divided into training (n = 924) and validation (n = 394) groups with a ratio of 7:3 (**Table S1**). Subsequently, six variables, including T stage, race, breast subtype, liver metastasis, lung metastasis, and radiotherapy, were determined as significant surgery-benefit-related factors based on univariate and multivariate logistics regression analysis (**Table 4**). Then, we established a predictive model as a visualized nomogram incorporating independent predictors to identify which BC patients with BM probably benefited from surgical resection of the primary lesion (**Figure 6**). We further developed the online version of this nomogram, which can be accessible at https://sunshine1.shinyapps.io/DynNomapp to facilitate its clinical application. The AUC value was 0.673 in the training cohort, 0.640 in the validation cohort, and 0682 in the external validation cohort, indicating the discrimination of this nomogram (**Figures 7**, **8A**). The calibration curves of the three cohorts presented that the actual observation results perfectly agreed with the nomogram-predicted values (**Figures 9**, **8B**). The DCA analysis displayed the good clinical utility of the model and suggested this nomogram could be a useful tool to identify optimal candidates for locoregional surgery clinically (**Figures 10**, **8C**).

**Table 4:** Univariate and multivariate logistic analysis for identifying surgical benefit-related variables in BC patients with BM.

Variables	Univariate analysis			Multivariate analysis		
	OR	95%CI	p-value	OR	95%CI	p-value
**Age**						
< 60 years	Reference					
≥ 60 years	0.757	0.584-0.981	0.036			
**Race**						
African American	Reference			Reference		
Other	1.548	0.857-2.796	0.148	1.412	0.765-2.605	0.270
Caucasian populations	2.128	1.482-3.057	<0.001	2.017	1.377-2.954	<0.001
**Sex**						
Female	Reference					
Male	3.702	0.743-18.441	0.110			
**Laterality**						
Left	Reference					
Right	0.857	0.661-1.111	0.245			
**Grade**						
I	Reference					
II	0.795	0.499-1.268	0.336			
III	0.469	0.295-0.748	0.001			
IV	0.002	0.001-0.003	1.000			
**T stage**						
T1-2	Reference			Reference		
T3-4	0.674	0.519-0.875	0.003	0.736	0.559-0.970	0.029
**N stage**						
N0	Reference					
N1-3	0.778	0.564-1.074	0.127			
**Primary site**						
Central portion of breast	Reference					
Lower-inner	1.053	0.453-2.444	0.905			
Lower-outer	1.228	0.568-2.653	0.601			
Upper-inner	0.992	0.518-1.902	0.982			
Upper-outer	0.847	0.510-1.406	0.520			
Other	0.795	0.491-1.286	0.349			
**Histology**						
Ductal	Reference					
Lobular	0.995	0.661-1.497	0.980			
Other	0.752	0.505-1.121	0.162			
**Breast subtype**						
HR+/HER2-	Reference			Reference		
HR+/HER2+	1.301	0.898-1.885	0.164	1.571	1.065-2.318	0.023
HR-/HER2+	0.657	0.378-1.142	0.137	0.948	0.531-1.694	0.858
HR-/HER2-	0.184	0.102-0.331	<0.001	0.234	0.128-0.427	<0.001
**Tumor size**						
< 5 cm	Reference					
5-10 cm	0.722	0.546-0.955	0.022			
> 10 cm	0.437	0.249-0.766	0.004			
**Lung metastasis**						
No	Reference					
Yes	0.703	0.506-0.978	0.036			
**Liver metastasis**						
No	Reference			Reference		
Yes	0.483	0.336-0.696	<0.001	0.510	0.346-0.752	0.001
**Brain metastasis**						
No	Reference			Reference		
Yes	0.457	0.218-0958	0.038	0.400	0.179-0.894	0.026
**Surgery to DM**						
No	Reference					
Yes	1.447	0.761-2.754	0.260			
**Radiotherapy**						
No	Reference			Reference		
Yes	1.373	1.052-1.792	0.020	1.527	1.147-2.032	0.004
**Chemotherapy**						
No	Reference					
Yes	0.994	0.765-1.291	0.961			

**Figure 11:**
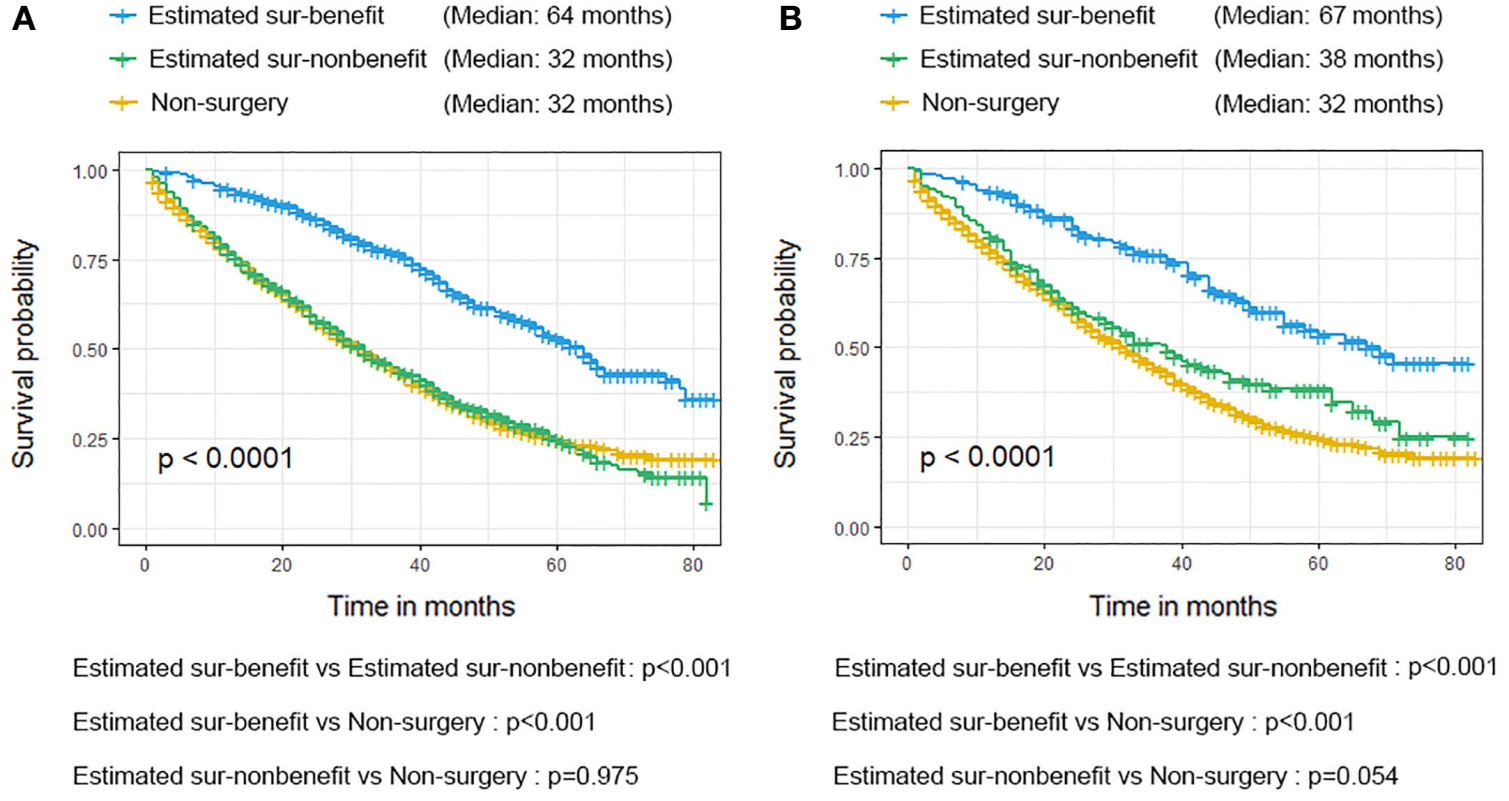
Validation of the nomogram in the training cohort **(A)** and validation cohort **(B)** using the Kaplan-Meier (K-M) survival analysis of the patients in the estimated sur-benefit subset, estimated sur-non-benefit subset, and Non-surgery group.

Additionally, we conducted the K-M survival analysis with a log-rank test in the matched cohort to verify the distinguishability of the newly proposed nomogram. As mentioned, 608 patients were assigned to the estimated sur-benefit subset, while the remaining 710 patients were assigned to the estimated sur-non-benefit subset. The K-M result exhibited that patients in the estimated sur-benefit subset had better survival outcomes than those in the Non-surgical group (p < 0.001) and the estimated sur-non-benefit subset (p < 0.001), both in the validation and training cohorts. However, no significant survival differences were observed between those patients who did not undergo surgery and those who were not estimated to benefit from surgery (all p > 0.05), indicating that BC patients with BM who were considered not to benefit from surgery according to our model did not improve their prognosis effectively even if they underwent surgery at the primary tumor site (**Figure 11**).

## Discussion

BC is one of the most prevalent malignancies worldwide, and tumor invasion and distant metastases are the leading causes of mortality in BC patients (16). Bone is the most frequently involved site in patients with metastatic BC, with approximately 65–75% of deceased BC patients carrying evidence of BM (17). To date, anti-tumor systemic therapy and bone-targeted drugs have been used to slow bone resorption and reduce the risk of bone-related events in BC patients with BM. Surgical resection of the primary lesion is not usually the preferred treatment option for these patients (18). However, recent retrospective studies have demonstrated that locoregional surgery has a significant survival advantage in BC patients with BM (11, 12, 19). The opinions from the 5th International Consensus Conference on Advanced Breast Cancer suggested that performing breast surgery in a select group of patients with metastatic BC may help improve quality of life and potentially prolong survival time. Continuing locoregional surgery has the potential to eliminate BC stem cells, decrease the tumor burden *in vivo*, improve the efficacy of systemic chemotherapy, and reduce the likelihood of primary tumor cells spreading to distant organs and forming new metastases (20, 21). In contrast, several researchers disagreed; previous studies concluded that surgical intervention on primary tumors did not improve the survival outcome of patients with metastatic BC, including bone metastases (8, 22). These slightly contradictory findings indicate that not all BC patients with BM could extend their survival with additional local surgery, creating a dilemma for surgeons. Nevertheless, existing guidelines fail to specify which BC patients with BM were suitable for surgical resection of the primary tumor. It remains an open question to determine which BC patients with BM would benefit from this procedure.

To address this concern, we systematically investigated the impact of local surgery on survival in BC patients with BM using the research data extracted from a population-based database. We developed a novel prediction model specifically for this population to quantify the probability of surgical benefit individually. Consistent with previous reports (11, 12, 23), our study revealed the potential of surgical resection of the primary tumor in managing BC patients with BM, suggesting local surgery could be a reasonable option in multimodality treatment to improve prognosis. Furthermore, the well-validated prediction model was expected to accurately stratify BC patients with BM according to their predicted potential to benefit from local surgery. The surgeons can more accurately identify the optimal surgical candidates with the assistance of this newly proposed nomogram, possibly allowing aggressive surgical treatment of the primary lesion to be more judiciously administered in BC patients with BM. Our analysis also suggested that local surgery did not improve survival in BC patients with BM estimated by our model as not benefiting from this surgical procedure (**Figure 8**). This group of patients may suffer from severe complications following invasive surgical procedures (24).

This study indicated that the probability of benefiting from local surgery for BC patients with BM was associated with six independent indicators, including race, T stage, breast subtype, liver metastasis, brain metastasis, and radiotherapy, among which the breast subtype illustrated the strongest correlation with the potential of surgical benefit. A large cohort study implemented by Tan and colleagues (25) suggested that patients with hormone receptor-positive (HR-positive) were more likely to benefit from surgical treatment of primary lesions than their counterparts with hormone receptor-negative (HR-negative). They examined 10,441 eligible metastatic BC patients from 2004 to 2008. They divided them into four groups: the primary and metastatic resection group (R0 group), the primary resection-only group, the metastatic resection-only group, and the non-surgery group. The median survival for these patients in four groups was 66, 52, 38, and 28 months, respectively (p < 0.05). In contrast, no statistically significant difference in survival was observed among the four surgical treatment groups in patients with HR-negative. Similarly, Neuman HB et al. reported a trend toward improved survival with primary tumor resection. It was noted most strongly in patients with ER/PR positive and/or HER2 positive disease. According to our model, patients with HR-/HER2- are least likely to benefit from surgical resection of the primary tumor. Additionally, we performed subgroup analyses to explore the heterogeneity in the treatment effect of local surgery. The results demonstrated that triple-negative breast cancer (TNBC) was the only subtype that may not benefit from surgery. TNBC is a highly aggressive subtype of BC characterized by extensive visceral metastases and frequent early postoperative recurrences (26, 27). TNBC patients with BM have a 40% lower five-year survival rate than non-TNBC patients (28). This suggests that effective systemic therapy may be even more significant for highly aggressive TNBC patients with BM. Besides, the status of liver metastasis and brain metastasis were also independently related to the surgical benefit in BC patients with BM. Previous studies have revealed that the prognosis of BC patients with BM may vary with the metastatic pattern (18, 29, 30). Two meta-analyses reported that patients with fewer metastases could benefit more substantially from local surgery (31, 32). Additionally, patients with brain and liver metastases were more severely ill than those with bone and lung metastases (33, 34). Likewise, our study suggested that in the case of the metastatic pattern of bone, liver, and brain metastases as well as bone, liver, lung, and brain metastases, surgery failed to provide survival benefit in these two subgroups. This suggests that BC patients with BM presenting with liver or brain metastases at initial presentation will have a poor prognosis, and their survival time will become limited, echoing the fact that the status of brain and liver metastases is closely relevant to whether patients can benefit from surgery at the primary site.

Notably, differences in sociodemographic factors and differentially expressed genes between white person and the African America patients may contribute to their differential survival benefit from locoregional surgery (35, 36). Meanwhile, the higher T stage usually represented more aggressive biological behavior and larger tumor size, which also were generally accompanied by abundant neovascularization; in such situations, extrusion during surgery would tremendously increase the risk of hematogenous metastases (37). Moreover, radiotherapy (RT) was considered a powerful adjunct therapy on perioperatively tumor control in BC patients with BM. A recent systematic review on treating BC patients with BM suggested that RT could decrease the local tumor burden in more radiosensitive subtypes and reduce the risk of local recurrence after surgery (38). Local control rates were higher in patients who received both local surgery and RT, indicating that RT improved quality of life and enhanced the survival benefit of local surgery (39, 40). Although patients treated with concomitant RT can better benefit from local surgery, the toxicity of radiotherapy also deserves attention. Chest radiation therapy may cause serious heart complications, such as premature coronary artery disease, valvular heart disease, pericarditis, arrhythmias, and restrictive/constrictive cardiomyopathy with heart failure (41). The newly proposed prediction model incorporating the above-mentioned independent predictors promises to be a useful tool for individualized quantification of the probability of benefiting from surgical resection of primary lesions in BC patients with BM and assisting in identifying optimal candidates for this procedure in clinical practice.

Nevertheless, this study still had several potential limitations. First, the study was retrospective and may have been subjected to selection bias related to the study design. Second, the SEER database lacked specific information about systemic treatments such as HER2 targeted therapy, endocrine therapy, and immunotherapy, so it is unclear whether locoregional surgery combined with these therapies could result in additional survival benefits. Besides, the SEER database failed to record some important information, including patient’s general condition and if they suffered complications, which may bias the choice of surgical treatment for the patients. Finally, further validation of our data is required in large-scale prospective studies.

## Conclusion

In this study, we thoroughly investigated the effect of local surgery on survival in BC patients with BM. We discovered that patients in specific groups could gain the survival benefit from this procedure. Additionally, a novel prediction model was proposed to quantify the probability of surgical benefit, allowing further selection of BC patients with BM suitable for surgical resection of primary tumors.

## Data availability statement

The raw data supporting the conclusions of this article will be made available by the authors, without undue reservation.

## Ethics statement

We received permission to access the research data file in the SEER program from the National Cancer Institute, US (reference number 15685-Nov2020). Approval was waived by the ethics committee of China-Japan Union Hospital of Jilin University, as SEER data is publicly available and de-identified.

## Author contributions

YT: Conceptualization, Data curation, Formal Analysis, Investigation, Methodology, Project administration, Resources, Software, Supervision, Validation, Visualization, Writing – original draft, Writing – review & editing. SX: Data curation, Methodology, Software, Writing – original draft. LJ: Formal Analysis, Project administration, Visualization, Writing – original draft. CZ: Data curation, Methodology, Writing – original draft. DZ: Conceptualization, Supervision, Writing – review & editing.
