# Supplementary material for: A visualized model for identifying optimal candidates for aggressive locoregional surgical treatment in patients with bone metastases from breast cancer
Source: Front Endocrinol (Lausanne). 2023 Oct 5;14:1266679. doi: 10.3389/fendo.2023.1266679 (PMC10585269; doi:10.3389/fendo.2023.1266679)
Supplement: Supplementary file 1 [file Table_1.docx]

**Table S1 The baseline data of BC patients with BM receiving surgery.**

| **Variables** | **Total**  **set**  **(**N=1318**)** | | **Training**  **set**  **(**N=924**)** | | **Validation**  **set**  **(**N=394**)** | | **External validation set**  (N=39) | | **p-value^#^** |
| --- | --- | --- | --- | --- | --- | --- | --- | --- | --- |
|  | **N** | **(%)** | **N** | **(%)** | **N** | **(%)** | **N** | **(%)** |  |
| **Age** |  |  |  |  |  |  |  |  | 0.5469 |
| ＜ 60 years | 664 | (50.38) | 460 | (49.78) | 204 | (51.78) | 21 | (53.85) |  |
| ≥ 60 years | 654 | (49.62) | 464 | (50.22) | 190 | (48.22) | 18 | (46.15) |  |
| **Race** |  |  |  |  |  |  |  |  | 0.5697 |
| African American | 226 | (17.15) | 165 | (17.86) | 61 | (15.48) | 39 | (100.0) |  |
| Other | 96 | (7.28) | 66 | (7.14) | 30 | (7.61) | 0 | (0) |  |
| Caucasian populations | 996 | (75.57) | 693 | (75.00) | 303 | (76.90) | 0 | (0) |  |
| **Sex** |  |  |  |  |  |  |  |  | 0.4403 |
| Female | 1304 | (98.94) | 916 | (99.13) | 388 | (98.48) | 39 | (100.0) |  |
| Male | 14 | (1.06) | 8 | (0.87) | 6 | (1.52) | 0 | (0) |  |
| **Primary site** |  |  |  |  |  |  |  |  | 0.1729 |
| Central portion | 113 | (8.57) | 78 | (8.44) | 35 | (8.88) | 2 | (5.13) |  |
| Lower-inner | 48 | (3.64) | 30 | (3.25) | 18 | (4.57) | 3 | (7.69) |  |
| Lower-outer | 68 | (5.16) | 39 | (4.22) | 29 | (7.36) | 3 | (7.69) |  |
| Upper-inner | 96 | (7.28) | 68 | (7.36) | 28 | (7.11) | 1 | (2.56) |  |
| Upper-outer | 366 | (27.77) | 258 | (27.92) | 108 | (27.41) | 9 | (23.08) |  |
| Other | 627 | (47.57) | 451 | (48.81) | 176 | (44.67) | 21 | (53.85) |  |
| **Grade** |  |  |  |  |  |  |  |  | 0.5687 |
| Grade I | 118 | (8.95) | 87 | (9.42) | 31 | (7.87) | 2 | (5.13) |  |
| Grade II | 585 | (44.39) | 401 | (43.40) | 184 | (46.70) | 22 | (56.41) |  |
| Grade III | 613 | (46.51) | 435 | (47.08) | 178 | (45.18) | 15 | (38.46) |  |
| Grade IV | 2 | (0.15) | 1 | (0.11) | 1 | (0.25) | 0 | (0) |  |
| **Laterality** |  |  |  |  |  |  |  |  | 0.1516 |
| Left | 671 | (50.91) | 458 | (49.57) | 213 | (54.06) | 18 | (46.15) |  |
| Right | 647 | (49.09) | 466 | 50.43 | 181 | (45.94 | 21 | (53.85) |  |
| **T stage** |  |  |  |  |  |  |  |  | 0.4892 |
| T1-T2 | 695 | (52.73) | 481 | (52.06) | 214 | (54.31) | 16 | (41.03) |  |
| T3-T4 | 623 | (47.27) | 443 | (47.94) | 180 | (45.69) | 23 | (58.97) |  |
| **N stage** |  |  |  |  |  |  |  |  | 0.8360 |
| N0 | 268 | (20.33) | 186 | (20.13) | 82 | (20.81) | 7 | (17.95) |  |
| N1-3 | 1050 | (79.67) | 738 | (79.87) | 312 | (79.19) | 32 | (82.05) |  |
| **Surgery to DM** |  |  |  |  |  |  |  |  | 1.0000 |
| No | 1263 | (95.83) | 885 | (95.78) | 378 | (95.94) | 37 | (94.87) |  |
| Yes | 55 | (4.17) | 39 | (4.22) | 16 | (4.06) | 2 | (5.13) |  |
| **Radiotherapy** |  |  |  |  |  |  |  |  | 0.1701 |
| No | 795 | (60.32) | 569 | (61.58) | 226 | (57.36) | 29 | (74.36) |  |
| Yes | 523 | (39.68) | 355 | (38.42) | 168 | (42.64) | 10 | (25.64) |  |
| **Chemotherapy** |  |  |  |  |  |  |  |  | 0.6009 |
| No | 576 | (43.70) | 399 | (43.18) | 177 | (44.92) | 18 | (46.15) |  |
| Yes | 742 | (56.30) | 525 | (56.82) | 217 | (55.08) | 21 | (53.85) |  |
| **Brain metastasis** |  |  |  |  |  |  |  |  | 0.8881 |
| No | 1268 | (96.21) | 888 | (96.10) | 380 | (96.45) | 38 | (97.44) |  |
| Yes | 50 | (3.79) | 36 | (3.90) | 14 | (3.55) | 1 | (2.56) |  |
| **Liver metastasis** |  |  |  |  |  |  |  |  | 0.8148 |
| No | 1094 | (83.00) | 765 | (82.79) | 329 | (83.50) | 36 | (92.31) |  |
| Yes | 224 | (17.00) | 159 | (17.21) | 65 | (16.50) | 3 | (7.69) |  |
| **Lung metastasis** |  |  |  |  |  |  |  |  | 0.6259 |
| No | 1058 | (80.27) | 738 | (79.87) | 320 | (81.22) | 35 | (89.74) |  |
| Yes | 260 | (19.73) | 186 | (20.13) | 74 | (18.78) | 4 | (10.26) |  |
| **Breast subtype** |  |  |  |  |  |  |  |  | 0.3975 |
| HR+/HER2- | 914 | (69.35) | 633 | (68.51) | 281 | (71.32) | 25 | (64.10) |  |
| HR+/HER2+ | 199 | (15.10) | 137 | (14.83) | 62 | (15.74) | 10 | (25.64) |  |
| HR-/HER2+ | 78 | (5.92) | 58 | (6.28) | 20 | (5.08) | 2 | (5.13) |  |
| HR-/HER2- | 127 | (9.64) | 96 | (10.39) | 31 | (7.87) | 2 | (5.13) |  |
| **Tumor size** |  |  |  |  |  |  |  |  | 0.3118 |
| ＜ 5 cm | 783 | (59.41) | 539 | (58.33) | 244 | (61.93) | 22 | (56.41) |  |
| 5-10 cm | 451 | (34.22) | 321 | (34.74) | 130 | (32.99) | 12 | (30.77) |  |
| ＞ 10 cm | 84 | (6.37) | 64 | (6.93) | 20 | (5.08) | 5 | (12.82) |  |
| **Histology** |  |  |  |  |  |  |  |  | 0.7055 |
| Ductal | 996 | (75.57) | 699 | (75.65) | 297 | (75.38) | 31 | (79.49) |  |
| Lobular | 158 | (11.99) | 107 | (11.58) | 51 | (12.94) | 5 | (12.82) |  |
| Other | 164 | (12.44) | 118 | (12.77) | 46 | (11.68) | 3 | (7.69) |  |

**^#^The result of P value was derived from the chi-square test for training set and testing set.**
